# Evaluation of in vitro drug-drug interactions of ivermectin and antimalarial compounds

**DOI:** 10.1186/s12936-025-05516-1

**Published:** 2025-09-24

**Authors:** Phornpimon Tipthara, Rattawan Kullasakboonsri, Kevin C. Kobylinski, Joel Tarning

**Affiliations:** 1 Mahidol Oxford Tropical Medicine Research Unit, Faculty of Tropical Medicine, Mahidol University, Bangkok, Thailand; 2 Centre for Tropical Medicine and Global Health, Nuffield Department of Clinical Medicine, University of Oxford, Oxford, UK

**Keywords:** Ivermectin, Microsomes, Metabolism, Antimalarial drugs

## Abstract

**Background:**

Ivermectin is lethal to *Anopheles* mosquitoes and a novel approach to malaria transmission control. Ivermectin could be co-administered with antimalarial drugs in mass drug administration, seasonal malaria chemoprevention, or other chemoprevention approaches. Co-administration with antimalarial drugs may impact ivermectin metabolism and/or absorption, resulting in increased or decreased exposure to ivermectin.

**Methods:**

To evaluate potential CYP-mediated drug-drug interactions (DDIs), ivermectin (1 µM) was incubated with pooled human liver microsomes, with and without the most commonly used antimalarial drugs at concentrations approximating twofold to tenfold the peak concentrations achieved following standard treatment. The antimalarial drugs investigated were dihydroartemisinin, piperaquine, chloroquine, artesunate, pyronaridine, mefloquine, artemether, lumefantrine, primaquine, atovaquone, proguanil, tafenoquine, sulfadoxine, pyrimethamine, and amodiaquine. Samples (50 µL) were collected at 0, 15, 30, 45, 60, 90, 120, and 150 min of incubation and ivermectin concentrations were measured using liquid chromatography-mass spectrometry. The metabolism rate of ivermectin was evaluated based on the normalized peak area (%) of ivermectin over a total of 150 min of incubation, applying linear regression to derive the rate of metabolism. Antimalarial compounds resulting in notable impact on the rate of ivermectin metabolism with a relative difference ≥ 50% and ≥ 25% were considered to have a substantial and partial effect on the in vitro metabolism of ivermectin, respectively.

**Results:**

Compounds that had a substantial DDI effect on the in vitro metabolism of ivermectin included piperaquine (98%), mefloquine (91%), chloroquine (76%), proguanil (60%), and lumefantrine (51%). Compounds that a partial DDI effect on the in vitro metabolism of ivermectin included atovaquone (48%), artesunate (27%), and pyronaridine (25%). All other antimalarials evaluated showed an in vitro interaction of 8–23%.

**Conclusions:**

Several of the commonly used antimalarial drugs, are mostly or in part metabolized by CYP3A4 and showed a notable DDI effect on the in vitro metabolism of ivermectin. This could potentially lead to clinically important pharmacokinetic and pharmacodynamic DDIs if co-administered, and needs to be evaluated in prospective clinical trials.

**Supplementary Information:**

The online version contains supplementary material available at 10.1186/s12936-025-05516-1.

## Background

Ivermectin mass drug administration (MDA) is under consideration as a novel vector control tool to suppress malaria parasite transmission due to its lethal effects on *Anopheles* mosquitoes [1]. Ivermectin could be combined with antimalarial drugs in MDAs to clear the human population of malaria parasites while simultaneously suppressing transmission by the extant mosquito population. Human clinical trials with ivermectin and dihydroartemisinin-piperaquine [2] plus single low-dose primaquine [3] or artemether-lumefantrine [4] have demonstrated the safety of these combinations. Indeed, MDAs with ivermectin and dihydroartemisinin-piperaquine have been performed at scale in The Gambia [5] and Guinea Bissau [6]. Co-administration of ivermectin and dihydroartemisinin-piperaquine increased the total exposure to ivermectin by 33%, leading to a 50% increase in mosquito mortality when blood was ingested 10 days post-treatment [3]. This increase in ivermectin mosquito-lethal effects would improve MDA impact, and thus warrants further evaluation to determine if other antimalarial compounds may have similar drug-drug interaction (DDI) with ivermectin.

Mass drug administrations with chloroquine, sulfadoxine-pyrimethamine, amodiaquine, primaquine, artesunate-amodiaquine, and dihydroartemisinin-piperaquine, have been performed for malaria control [7–9]. Effective chemoprevention with mefloquine, tafenoquine, and atovaquone-proguanil [10] has been demonstrated. Seasonal Malaria Chemoprevention (SMC) with sulfadoxine-pyrimethamine plus amodiaquine is provided to millions of children across Africa. Ivermectin could be combined with MDA, SMC, or chemoprevention intervention to reduce *Plasmodium* transmission, which could limit antimalarial drug resistance development when implementing MDA, SMC or chemoprevention. Not all blood-stage antimalarial drugs effectively prevent transmission to mosquitoes, thus co-administration of ivermectin might reduce onwards transmission of potentially resistant parasites from the treated person. Furthermore, removing mosquitoes with parasites from the extant population of wild mosquitoes could limit transmission of parasites back to persons with potentially suboptimal blood level concentrations of antimalarial drugs, which is the most critical point when drug resistance is most likely to develop. Additionally, ivermectin co-administration would have a direct personal benefit for MDA recipients as ivermectin can treat and prevent a wide range of neglected tropical diseases. This study investigated the potential DDI of commonly used antimalarial drugs on ivermectin in vitro metabolism using human liver microsomes.

## Methods

### Chemicals and materials

Drug compounds (except sulfadoxine), pooled human liver microsomes, β-nicotinamide adenine dinucleotide 2′-phosphate reduced tetrasodium salt hydrate (β-NADPH), and ammonium acetate (LC–MS grade) were purchased from Sigma-Aldrich (St. Louis, MO, USA). Sulfadoxine was purchased from Biosynth Carbosynth (Berkshire, UK). Potassium phosphate 0.5 M buffer solution (pH 7.4) was purchased from Thermo Fisher Scientific (Waltham, MA, USA). Ivermectin-D2 was purchased from Toronto Research Chemicals (Toronto, ON, Canada). Formic acid (LC–MS grade) was purchased from Honeywell Fluka (Seelze, Germany). LC–MS grade acetonitrile and methanol were purchased from J.T. Baker (Phillipsburg, NJ, USA). Water (milli-Q 18.2 MΩ cm − 1) was prepared from a Milli-Q purification system (Merck, Darmstadt, Germany). Drug solutions were prepared as stated in Table S1. Most drugs were evaluated at approximately tenfold C_max_, the exceptions being dihydroartemisinin, sulfadoxine, atovaquone, and lumefantrine evaluated at two to threefold C_max_. The final drug concentrations of the microsome incubations and reported C_max_ values are shown in Table 1.

**Table 1:** Preparation of drugs used in the human liver microsome evaluations

Compound	Final conc. (µM)	Reported C_max_ (µM)	Reported C_max_ (ng/ml)
Dihydroartemisinin	5.0	1.48	421 [3]
Piperaquine	10.0	1.06	568 [11]
Chloroquine	40.0	4.47	1430 [11]
Artesunate	6.0	0.63	244 [11]
Pyronaridine	10.0	1.10	571 [12]
Mefloquine	70.0	7.14	2700 [11]
Artemether	6.0	0.62	186 [11]
Lumefantrine	80.0	47.27	25,700 [11]
Primaquine	6.0	0.64	167 [11]
Atovaquone	60.0	36.26	13,300 [11]
Proguanil	30.0	2.96	750 [11]
Tafenoquine	6.0	0.65	300 [13]
Sulfadoxine	800.0	418.91	130,000 [11]
Pyrimethamine	10.0	1.13	280 [11]
Amodiaquine	0.4	0.04	15.5 [11]
Ivermectin	1.0	0.14	119 [3]

Final conc. is the final antimalarial drug concentration in the microsome incubation, and Reported C_max_ is the reported peak concentration in a patient population associated with standard dosing

### Microsome assay

Pooled human liver microsomes (containing 20 mg/mL protein) were thawed on ice, and the reactions were conducted in a 96-well plate (1 mL, Agilent Technologies, Santa Clara, CA, USA). The assay buffer consisted of 0.1 M potassium phosphate buffer (pH 7.4). The premix solutions for each condition were prepared with specific volumes of each component, as outlined in Table S1. The premix solution was prepared by combining ivermectin, antimalarial drug, microsomes, and buffer. Three replicates of each premix solution were prepared, and 475 µL of each premix was transferred into separate wells of the 96-well plate. The plate was incubated at 37 °C for 5 min with gentle shaking (300 rpm). Following incubation, 25 µL of 20 mM NADPH was added to each well, and the solution was mixed thoroughly. The final incubation mixture (500 µL) contained a total microsome concentration of 0.53 mg/mL. To monitor the metabolism of ivermectin, 50 µL from each well was collected at 0, 15, 30, 45, 60, 90, 120, and 150 min and transferred to a new plate containing 200 µL of cold acetonitrile and 100 ng/mL of ivermectin-D2 (internal standard). The collection plate was kept on ice during sampling. After sample collection, the plate was sealed and centrifuged at 1,100 × *g* for 15 min at 4 °C. A 100 µL aliquot of the clear supernatant was transferred to a new plate for immediate LC–MS analysis.

### Liquid chromatography-mass spectrometry analysis

The LC system consisted of an Agilent 1260 quaternary pump, an Agilent 1260 autosampler (set at 6 °C), and an Agilent 1290 column compartment (set at 40 °C). Five µL of sample was analysed on an Acquity UPLC HSS T3 reversed-phase column (2.1 × 100 mm, 1.8 μm) with a precolumn (2.1 × 5 mm, 1.8 μm) under a linear gradient. The mobile phase consisted of water with 10 mM ammonium acetate and 0.1% formic acid (A) and acetonitrile:water (95:5, v/v) with 10 mM ammonium acetate and 0.1% formic acid (B), at a flow rate of 0.3 mL/min. The gradient started at 75% B, ramped to 90% B over 2 min, held at 90% B for 4 min, and returned to 75% B in 0.1 min, with a 3.9-min re-equilibration. A Sciex TripleTOF 5600 + Q-TOF MS with DuoSpray ESI was used for LC–MS analysis in the positive mode, with source conditions set at 40 psi for ion source gas 1 (GS1) and gas 2 (GS2), 30 psi for curtain gas (CUR), 4,500 V for ion spray voltage floating (ISVF), 350 °C source temperature (TEM), and 120 V declustering potential (DP). Data were acquired from m/z 100–1,000 in TOF–MS scan mode using Analyst TF Software 1.8, and ivermectin quantification (ivermectin peak area/internal standard peak area) was performed with MultiQuant Software (Sciex, Framingham, MA, USA) with a high resolution TOF–MS scan mode.

### Data analysis

The metabolism rate of ivermectin was evaluated based on the normalized peak area (%) of ivermectin over a total of 150 min of incubation. The observed peak area of ivermectin was normalized to the peak area of the internal standard, ivermectin-d2, for each individual measurement. This normalized value was further adjusted relative to the pre-incubation ivermectin peak area (zero-minute sample) within a sample collection series, resulting in final ivermectin concentrations expressed as relative peak areas. All individual ivermectin concentrations (relative peak area) from triplicate incubations were fitted to an ordinary linear regression, separately conducted for ivermectin alone and ivermectin co-incubated with an antimalarial. The estimated (±95% CI) slope and intercept of the regression analysis was derived using GraphPad Prism v.10.4.2. Additionally, the slopes of the regression analyses were compared statistically between ivermectin alone and when co-incubation with an antimalarial, as an automated output from the regression analysis. Furthermore, the relative difference (%) in in vitro metabolism rate (i.e. difference between regression slopes) for ivermectin alone and when co-incubated with an antimalarial was calculated as shown in Eq. 1. The 95% confidence interval of the calculated relative difference was approximated by propagation of the relative standard errors of the individual slope estimates (Eqs. 2, 3).

1 $$Relative\, difference \left(RD\right)=\frac{\left|A-B\right|}{\left|A\right|}$$

2 $${Standard\, error (SE}_{RD})=\sqrt{{{\left(\frac{-\left(A-B\right)}{{A}^{2}}\times {SE}_{A}\right)}^{2}+\left(\frac{1}{A}\times {SE}_{B}\right)}^{2}}$$

3 $$95\% confidence\, interval (95\% CI)=RD\pm {1.96\times SE}_{RD}$$

where, A is the slope of the regression analysis of ivermectin when incubated alone, B is the slope of the regression analysis of ivermectin when co-incubated with an antimalarial, SE_A_ is the standard error of slope A, SE_B_ is the standard error of slope B, and SE_RD_ is the approximated standard error of the relative difference. Any co-incubation resulting in a relative difference greater than an assigned arbitrary value of 50% and 25% were deemed to have a substantial and partial DDI affecting the in vitro metabolism of ivermectin, respectively.

## Results

**Fig. 1:**
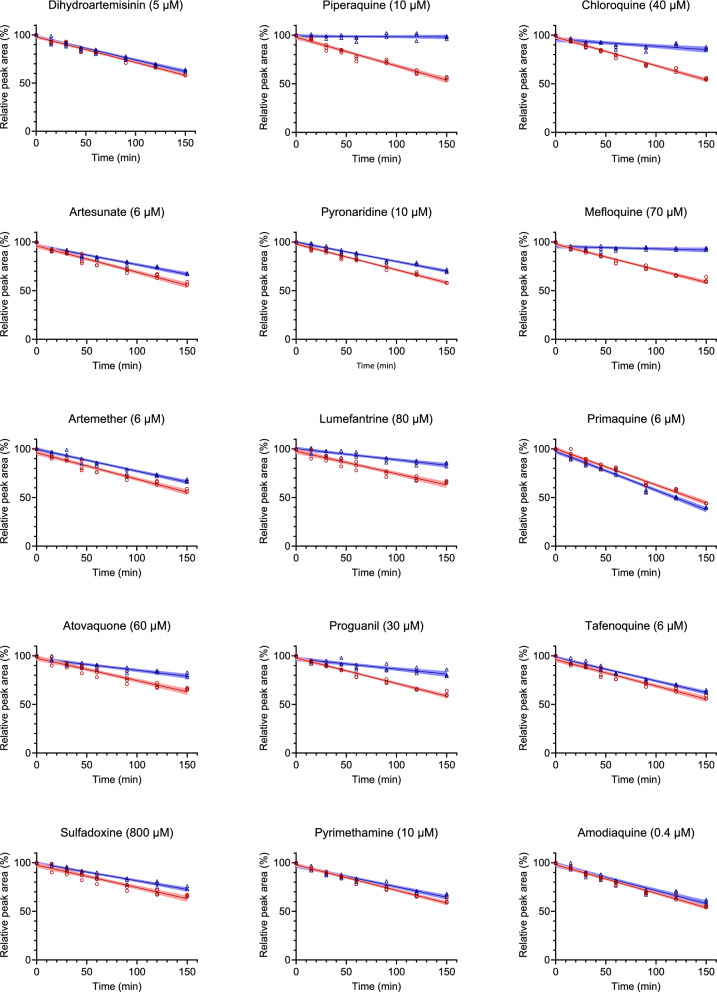
In vitro metabolism of ivermectin when incubated alone (red circles) and in combination with various antimalarial compounds (blue triangles), using pooled human liver microsomes. Drug concentrations are in micromolar (µM). Ivermectin peak areas were normalized to the internal standard (ivermectin-d_2_), then further normalized to the zero-minute sample to yield relative peak areas. Observed data (open markers) display triplicate biological measurements. Solid lines depict linear regression based on observed data, and shaded areas show the 95% prediction interval of the linear regression analysis

Piperaquine, mefloquine, chloroquine, proguanil, and lumefantrine showed a substantial DDI by reducing the in vitro metabolism rate of ivermectin. Atovaquone, artesunate, and pyronaridine showed a partial DDI by reducing the in vitro metabolism rate of ivermectin. While sulfadoxine, pyrimethamine, artemether, tafenoquine, amodiaquine, dihydroartemisinin, and primaquine had little impact on the in vitro metabolism of ivermectin (Fig. 1; Table 2, and Table S2).

**Table 2:** Relative difference in in vitro metabolism of ivermectin when incubated alone and with an antimalarial compound

Compound	Relative difference (95% CI)	p-value	Metabolism pathway
Piperaquine	98.1% (87.7–108.5)	< 0.001	Major CYP3A4; Minor CYP2C8 [14]
Mefloquine	90.6% (80.4–100.8)	< 0.001	CYP3A4 [15, 16]
Chloroquine	76.4% (66–86.8)	< 0.001	Major CYP3A4, CYP2C8; Minor CYP2D6 [17]
Proguanil	60% (49.6–70.5)	< 0.001	CYP2C19 [18]
Lumefantrine	50.9% (40.2–61.6)	0.0028	CYP3A4 [19, 20]
Atovaquone	47.6% (36.7–58.6)	0.0043	Major CYP3A4; Minor CYP2C9 [21, 22]
Artesunate	26.9% (19.8–34)	< 0.001	Major CYP2A6; Minor CYP2B6 [23]
Pyronaridine	25.1% (19.5–30.8)	< 0.001	CYP1A2, CYP2D6, CYP3A4 [24]
Sulfadoxine	22.5% (14.3–30.8)	0.0595	Acetylation by N-Acetyltransferase 2 [25]
Pyrimethamine	17.8% (9.9–25.8)	0.0797	CYP1B1, CYP2C9 [23]
Artemether	17% (10.1–23.9)	0.0058	Major CYP2B6; Minor CYP3A4 [19, 20, 26]
Tafenoquine	10.8% (4.2–17.4)	0.0705	CYP2D6 [27]
Amodiaquine	9.16% (− 0.71 to 19.04)	0.1269	Major CYP2C8; Minor CYP1A1, CYP1B1 [23, 28]
Dihydroartemisinin	9.09% (2.04–16.13)	0.0597	Glucuronidation by UGT1A9 and UGT2B7 [29]
Primaquine	7.72% (1.46–13.98)	0.073	Major CYP2D6; Minor CYP1A2 [23, 30]
Ivermectin	N/A	N/A	Major CYP3A4; Minor CYP3A5, CYP2C8 [31, 32]

## Discussion

Ivermectin is primarily metabolized by CYP3A4 and to a lesser extent CYP3A5 and CYP2C8 [31, 32]. Most compounds that showed a substantial interaction with ivermectin are metabolized primarily or in part by CYP3A4 (Fig. 1; Table 2). Co-administration of ivermectin and dihydroartemisinin-piperaquine in healthy volunteers in Thailand resulted in substantially increased exposure to ivermectin [3]. In vitro findings presented here indicate this is most likely due to a decreased metabolism of ivermectin on account of competitive enzyme inhibition by piperaquine, and with little interactions by dihydroartemisinin. Co-administration of ivermectin and chloroquine in Rhesus macaques resulted in increased exposure to ivermectin [33, 34]. This finding is supported here by the 76% decreased metabolism of ivermectin when incubated with chloroquine. Co-administration of ivermectin and artemether-lumefantrine was shown to be safe in a clinical trial, but its impact on ivermectin metabolism was not evaluated [4].

Lumefantrine, mefloquine, chloroquine, atovaquone, and pyronaridine are all metabolized by CYP3A4 and showed partial to substantial DDIs with ivermectin in this in vitro system and should be evaluated in prospective clinical trials before co-administered clinically. A likely explanation for this DDI is competitive inhibition of the metabolism of ivermectin by the antimalarial drug. The co-administered antimalarial drug might compete for the same enzymatic metabolism pathway, and depending on the difference in affinity of the drug molecules to the active site, the antimalarial drug could produce a varying degree of reduced enzymatic activity in the metabolism of ivermectin, resulting in slower metabolism, higher plasma concentrations, and a prolonged effect of ivermectin. Proguanil showed a substantial reduction in ivermectin metabolism (60%), which is somewhat surprising given that ivermectin is not metabolized by CYP2C19 [32]. However, limited studies on proguanil metabolism have been performed, restricted to one report from a pharmacogenomic study [18]. Thus, it is possible that proguanil is in part metabolized by CYP3A4 or that proguanil actively inhibits the activity of CYP3A4, which would explain the DDI observed here. Further in vitro assessment is warranted to identify specific CYPs involved in proguanil metabolism and the mechanism of this DDI. Drugs with minimal observed effects are primarily metabolized by other CYP enzymes than CYP3A4, the exception being artemether in which CYP3A4 is a minor contributor to metabolism.

It should be noted that microsome systems are highly effective in metabolizing drug molecules in vitro and results might over-estimate potential DDIs in vivo, and it is possible that a substantial in vitro DDI seen here might not result in a clinically relevant DDI when evaluated in patients. On the other hand, many of the compounds evaluated here are administered as combinations (e.g. artesunate-pyronaridine, artesunate-mefloquine, atovaquone-proguanil) and thus may have substantially greater impact on ivermectin metabolism in vivo than could be predicted in these single compound in vitro assay evaluations. Ivermectin has a very broad therapeutic window when used to treat neglected tropical diseases, with a very favorable safety profile. Thus, increasing the duration of time that ivermectin blood concentrations are high enough to produce mosquito-lethal effects, as observed with dihydroartemisinin-piperaquine coadministration [3], should improve overall control outcomes and be considered an added benefit of the co-administration.

Data generated here could also be used to develop, refine and inform physiologically-based pharmacokinetic (PBPK) models to predict potential in vivo DDIs in different populations and clinical scenarios. Developed and validated PBPK compound files are available for the most common antimalarials [35] and for ivermectin [36] and in vitro interaction data generated here could be incorporated to simulate concentration–time profiles of ivermectin in the presence of clinically relevant doses of antimalarial drugs. Such models could provide additional insight into the concomitant use of these drugs as it also takes into account the time-aspect of differing pharmacokinetic profiles and elimination half-lives of the evaluated drugs. Simulating full pharmacokinetic concentration–time profiles and linking these to pharmacodynamic models, under different concomitant drug scenarios would provide crucial information regarding the safety and therapeutic effects of these DDIs.

## Conclusions

Co-incubation of antimalarial drugs metabolized by CYP3A4 and ivermectin showed increased exposure to ivermectin due to competitive inhibition of the metabolism of ivermectin, when evaluated in pooled human liver microsomes. This could potentially result in clinically important DDIs if co-administered during MDA, SMC or chemoprevention, resulting in altered mosquito lethal effects of ivermectin. We suggest that this should be evaluated in prospective clinical trials before implementation on a larger scale.

## Supplementary Information

Additional file 1

## Data Availability

Data is available upon reasonable request to the authors.

## Abbreviations

MDAs: Mass drug administration
SMC: Seasonal Malaria Chemoprevention
LC–MS/MS: Liquid chromatography-mass spectrometry/mass spectrometry
C_max_: Peak concentration
DDI: Drug-drug interaction
