# Supplementary material for: Evaluation of in vitro drug-drug interactions of ivermectin and antimalarial compounds
Source: Malar J. 2025 Sep 24;24:290. doi: 10.1186/s12936-025-05516-1 (PMC12462173; doi:10.1186/s12936-025-05516-1)
Supplement: Supplementary file 1 — Additional file 1 [file 12936_2025_5516_MOESM1_ESM.docx]

**Supplement Material - Evaluation of drug-drug interactions of ivermectin and antimalarial compounds**

*Phornpimon Tipthara, Rattawan Kullasakboonsri, Kevin C. Kobylinski, Joel Tarning*

| **Table S1. Solutions and volumes used in microsomes assay reactions** | | | | | | | | | |
| --- | --- | --- | --- | --- | --- | --- | --- | --- | --- |
| **Compound** | **Solvent** | **Antimalarial solution** | |  | **Solution volumes (µL)** | | | | |
|  |  | **Stock conc. (mM)** | **Working conc.  (µM)** |  | **IVM working solution** | **Antimalarial working solution** | **HLM** | **NADPH** | **KPi** |
| IVM + Dihydroartemisinin | 80% ethanol | 7.74 | 500 |  | 5 | 5 | 12.5 | 25 | 452.5 |
| IVM + Piperaquine | 0.5% formic acid in methanol | 1.01 | 500 |  | 5 | 10 | 12.5 | 25 | 447.5 |
| IVM + Chloroquine | 80% acetonitrile | 5.23 | 500 |  | 5 | 40 | 12.5 | 25 | 452.5 |
| IVM + Artesunate | 80% ethanol | 6.5 | 200 |  | 5 | 15 | 12.5 | 25 | 442.5 |
| IVM + Pyronaridine | 80% methanol | 2.74 | 1000 |  | 5 | 5 | 12.5 | 25 | 452.5 |
| IVM + Mefloquine | 80% methanol | 5.55 | 700 |  | 5 | 50 | 12.5 | 25 | 407.5 |
| IVM + Artemether | 80% ethanol | 6.7 | 600 |  | 5 | 5 | 12.5 | 25 | 452.5 |
| IVM + Lumefantrine | Dimethyl sulfoxide | 20 | 8000 |  | 5 | 5 | 12.5 | 25 | 452.5 |
| IVM + Primaquine | 80% acetonitrile | 5.71 | 600 |  | 5 | 5 | 12.5 | 25 | 452.5 |
| IVM + Atovaquone | Dimethyl sulfoxide | 30 | 6000 |  | 5 | 5 | 12.5 | 25 | 452.5 |
| IVM + Proguanil | 80% methanol | 7.58 | 400 |  | 5 | 37.5 | 12.5 | 25 | 420 |
| IVM + Tafenoquine | 80% methanol | 3.95 | 600 |  | 5 | 5 | 12.5 | 25 | 452.5 |
| IVM + Sulfadoxine | 80% acetonitrile | 80 | 80000 |  | 5 | 5 | 12.5 | 25 | 452.5 |
| IVM + Pyrimethamine | 80% acetonitrile | 9.25 | 1000 |  | 5 | 5 | 12.5 | 25 | 452.5 |
| IVM + Amodiaquine | 80% acetonitrile | 5.59 | 40 |  | 5 | 5 | 12.5 | 25 | 452.5 |
| IVM alone | 80% acetonitrile | 3.2 | 100 |  | 5 | 0 | 12.5 | 25 | 457.5 |

*The final concentration of ivermectin was 1.0 µM in all incubations. Drug stock solutions were prepared in the solvent listed in the table. Drug working solutions were diluted in 80% acetonitrile in water, except lumefantrine and atovaquone were diluted in dimethyl sulfoxide. IVM is ivermectin, HLM is human liver microsomes, NAPDH is nicotinamide adenine dinucleotide phosphate, KPi is potassium phosphate buffer.*

| **Table S2. Slope, intercept and relative difference in *in vitro* metabolism of ivermectin when incubated alone and with an antimalarial compound** | | | | | |
| --- | --- | --- | --- | --- | --- |
| **Compound** | **Ivermectin alone** | | **Combination** | | **Relative difference (95%CI)** |
|  | **Slope (95%CI)** | **Intercept (95%CI)** | **Slope (95%CI)** | **Intercept (95%CI)** |  |
| Piperaquine | -0.29 (-0.32 to -0.27) | 97.8 (95.9-99.8) | -0.01 (-0.03 to 0.01) | 99.0 (97.4-100.6) | 98.1% (87.7-108.5) |
| Mefloquine | -0.26 (-0.28 to -0.24) | 97.7 (96.2-99.2) | -0.02 (-0.05 to 0.00) | 95.5 (93.8-97.1) | 90.6% (80.4-100.8) |
| Chloroquine | -0.29 (-0.31 to -0.28) | 98.0 (96.6-99.4) | -0.07 (-0.10 to -0.04) | 95.5 (93.3-97.7) | 76.4% (66.0-86.8) |
| Proguanil | -0.26 (-0.28 to -0.24) | 97.7 (96.2-99.2) | -0.10 (-0.13 to -0.08) | 96.9 (94.9-98.9) | 60.0% (49.6-70.5) |
| Lumefantrine | -0.23 (-0.26 to -0.20) | 97.6 (95.4-99.9) | -0.11 (-0.13 to -0.09) | 100.2 (98.6-101.8) | 50.9% (40.2-61.6) |
| Atovaquone | -0.23 (-0.26 to -0.20) | 97.6 (95.4-99.9) | -0.12 (-0.14 to -0.10) | 97.2 (95.5-98.9) | 47.6% (36.7-58.6) |
| Artesunate | -0.27 (-0.30 to -0.25) | 96.1 (94.1-98.1) | -0.20 (-0.22 to -0.18) | 96.6 (95.1-98.0) | 26.9% (19.8-34.0) |
| Pyronaridine | -0.27 (-0.28 to -0.25) | 98.0 (96.7-99.2) | -0.20 (-0.21 to -0.18) | 99.9 (98.7-101.0) | 25.1% (19.5-30.8) |
| Sulfadoxine | -0.23 (-0.26 to -0.20) | 97.6 (95.4-99.9) | -0.18 (-0.20 to -0.16) | 99.4 (97.9-100.8) | 22.5% (14.3-30.8) |
| Pyrimethamine | -0.26 (-0.28 to -0.24) | 97.7 (96.2-99.2) | -0.21 (-0.24 to -0.19) | 96.7 (95.0-98.3) | 17.8% (9.90-25.8) |
| Artemether | -0.27 (-0.30 to -0.25) | 96.1 (94.1-98.1) | -0.22 (-0.24 to -0.21) | 99.7 (98.2-101.1) | 17.0% (10.1-23.9) |
| Tafenoquine | -0.27 (-0.30 to -0.25) | 96.1 (94.1-98.1) | -0.24 (-0.26 to -0.22) | 98.4 (96.9-99.8) | 10.8% (4.20-17.4) |
| Amodiaquine | -0.29 (-0.31 to -0.28) | 98.0 (96.6-99.4) | -0.27 (-0.30 to -0.24) | 98.1 (95.7-100.4) | 9.16% (-0.71-19.04) |
| Dihydroartemisinin | -0.27 (-0.28 to -0.25) | 98.0 (96.7-99.2) | -0.24 (-0.26 to -0.22) | 98.1 (96.6-99.6) | 9.09% (2.04-16.13) |
| Primaquine | -0.37 (-0.39 to -0.35) | 100.1 (98.5-101.7) | -0.40 (-0.42 to -0.38) | 97.6 (95.7-99.4) | 7.72% (1.46-13.98) |
